# A functional Magnetic Resonance Imaging study of neurohemodynamic abnormalities during emotion processing in subjects at high risk for schizophrenia

**DOI:** 10.4103/0019-5545.74304

**Published:** 2010

**Authors:** Ganesan Venkatasubramanian, Dawn Thomas K. Puthumana, Peruvumba N. Jayakumar, B. N. Gangadhar

**Affiliations:** Department of Psychiatry, National Institute of Mental Health and Neuro Sciences (NIMHANS), Bangalore, Karnataka, India; 1 Department of Neuroimaging and Interventional Radiology, National Institute of Mental Health and Neuro Sciences (NIMHANS), Bangalore, Karnataka, India

**Keywords:** Functional magnetic resonance imaging, high-risk subjects, schizophrenia

## Abstract

**Background:**

Emotion processing abnormalities are considered among the core deficits in schizophrenia. Subjects at high risk (HR) for schizophrenia also show these deficits. Structural neuroimaging studies examining unaffected relatives at high risk for schizophrenia have demonstrated neuroanatomical abnormalities involving neo-cortical and sub-cortical brain regions related to emotion processing. The brain functional correlates of emotion processing in these HR subjects in the context of ecologically valid, real-life dynamic images using functional Magnetic Resonance Imaging (fMRI) has not been examined previously.

**Aim:**

To examine the neurohemodynamic abnormalities during emotion processing in unaffected subjects at high risk for schizophrenia in comparison with age-, sex-, handedness- and education-matched healthy controls, using fMRI.

**Materials and Methods:**

HR subjects for schizophrenia (*n*=17) and matched healthy controls (*n*=16) were examined. The emotion processing of fearful facial expression was examined using a culturally appropriate and valid tool for Indian subjects. The fMRI was performed in a 1.5-T scanner during an implicit emotion processing paradigm. The fMRI analyses were performed using the Statistical Parametric Mapping 2 (SPM2) software.

**Results:**

HR subjects had significantly reduced brain activations in left insula, left medial frontal gyrus, left inferior frontal gyrus, right cingulate gyrus, right precentral gyrus and right inferior parietal lobule. Hypothesis-driven region-of-interest analysis revealed hypoactivation of right amygdala in HR subjects.

**Conclusions:**

Study findings suggest that neurohemodynamic abnormalities involving limbic and frontal cortices could be potential indicators for increased vulnerability toward schizophrenia. The clinical utility of these novel findings in predicting the development of psychosis needs to be evaluated.

## INTRODUCTION

### Emotion processing deficits: A trait marker in schizophrenia

Schizophrenia is a complex neuropsychiatric disorder characterized by delusions, hallucinations, negative symptoms, and cognitive deficits.[1] Emotion processing abnormalities are among the important cognitive deficits in schizophrenia. Studies using facial emotion recognition task have shown schizophrenia patients to have significant Facial Emotion Recognition Deficits, indicating their impaired ability to perceive and process facial affective expressions; also, they have difficulties in expressing facial emotional expressions.[2–5]

Facial Emotion Recognition Deficits appear to be intrinsic to the pathogenesis of schizophrenia since this has been demonstrated even in antipsychotic-naïve patients.[6,7] Although these deficits are more severe during psychotic exacerbations, they are observed even in patients in whom the symptoms are under remission.[8] Facial Emotion Recognition Deficits are not correlated with duration of symptoms, demographic characteristics[9] or therapeutic effect of antipsychotics.[10] All these observations strongly support that these emotion-processing deficits could well be a trait marker for schizophrenia.

### Emotion processing deficits in schizophrenia: A separate dimension

The basis for these emotion recognition deficits in schizophrenia patients has been examined in the background of two contrasting views. According to one perspective, poor performance on affect perception tasks merely reflects a generalized performance deficit, such that schizophrenia patients perform about as poorly on affect perception tasks as they do on any given neurocognitive task.[7] An alternative proposal is that impaired performance of schizophrenia patients in recognizing emotions is a separate and specific deficit rather than being reflective of a generalized deficit. Many studies have supported this alternative proposal.[11–16] For example, it has been shown that affect perception deficits are associated with visual scanning deficits, but less strongly associated with vigilance and working memory.[16]

Importantly, these deficits have functional significance.[17] Emotion processing deficits result in significant social dysfunction in schizophrenia patients.[18] For example, studies have shown that facial affect recognition deficits are associated with dysregulated social behavior and interpersonal difficulties in schizophrenia.[17]

### Emotion processing deficits and vulnerability to schizophrenia

Since emotion processing deficits are trait markers intrinsic to the pathogenesis of schizophrenia and perhaps form a separate and specific dimension of cognitive deficit with significant impact on functional status of the patient, it might potentially be associated with vulnerability to develop schizophrenia. Previous clinical/behavioral studies[16,19–21] have examined for Facial Emotion Recognition Deficits in first-degree relatives of schizophrenia patients, who have a higher risk to develop this disorder. McCown *et al*.[19] examined parents of schizophrenia patients in comparison to parents of patients with medical or surgical diseases, using slides of faces selected from Ekman and Friesen’s Basic Affect Recognition Test.[22] The schizophrenia patients’ parents performed poorly than their controls. In another study, the first-degree non-psychotic relatives of schizophrenia patients performed worse than controls on the measure of social perception.[20] Recent studies have demonstrated unaffected biological siblings of schizophrenia patients to have subtle emotion processing deficits in comparison to matched healthy controls.[16,21] All these observations strongly support the “Facial Emotion Recognition Deficit” as a potential “endophenotype” in schizophrenia. Hence, systematic elucidation of the neurobiological basis of this endophenotype can help us to understand the pathogenesis of schizophrenia.

### Neural basis of Facial Emotion Recognition Deficits in HR subjects for schizophrenia

Functional neuroimaging studies examining the neural basis of emotion processing in healthy subjects have shown involvement of a network of brain regions (amygdala, hippocampus, prefrontal and parietal cortices).[23] Consistent with these studies, both neuroanatomical as well as neurofunctional imaging studies have demonstrated abnormalities in many of these brain regions in schizophrenia patients during emotion processing tasks.[24,25] However, the neural basis of facial emotion processing in unaffected subjects at high risk for schizophrenia (HR subjects) has not been examined adequately. To the best of our knowledge, only one functional Magnetic Resonance Imaging (fMRI) study has examined in non-affected brothers of schizophrenia patients (*N*=13) in which the HR subjects demonstrated hypoactivity of amygdale.[26] However, this study is limited by the following significant factors: 1) ‘Static versus dynamic emotional expression stimuli’: The emotion recognition stimuli used in this study consisted of slides showing photographs of various emotional expressions. However, this does not correspond to the real-life expression of emotions, which is continuous and dynamic. Indeed, it has been recommended that dynamic movie clips rather than static photo images need to be used in facial emotion recognition studies.[5] 2) ‘Ecological validity of the fMRI paradigm’: “Ecological validity” of an fMRI task refers to the extent to which the findings observed during the task performance of subjects within the MRI scanner can be generalizable to the environment. For an fMRI task to have “optimal ecological validity”, it has to be i) simple, ii) unlikely to be confounded by differential stimulus delivery (i.e. the active task as well as the neutral task should be well matched) and iii) should resemble the real-life situation to the closest possible extent.[27] The authors of the previous study have themselves mentioned that their study is limited by these fMRI task related methodological issues.[26]

In this study, using fMRI (a brief summary of the basic principles of fMRI has been mentioned in the “Materials and Methods” section) we examined the neurohemodynamic correlates of facial emotion recognition in unaffected healthy siblings who were at high risk for developing schizophrenia [high-risk (HR) subjects; *n*=17] in comparison to age-, sex-, handedness- and education-matched (as a group) healthy controls without high risk for developing schizophrenia [low-risk (LR) subjects; *n*=16]. To the best of our knowledge, this is the first study to examine HR subjects for schizophrenia utilizing an “ecologically valid” implicit facial emotion recognition task using dynamic images (movie clips) showing continuous emotional expressions (fear, happy, and neutral). Importantly, these dynamic images were derived from a validated tool culturally appropriate for Indian subjects, namely, the Tool for Recognition of Emotions in Neuropsychiatric Disorders (TRENDS).[28] We hypothesized that HR subjects will have hypoactivation of emotional brain regions (limbic brain regions, specifically amygdala and related network brain regions) in comparison to LR subjects.

## MATERIALS AND METHODS

### Selection of subjects

Seventeen “HR” biological siblings (brother or sister) of treatment-seeking patients with schizophrenia [Diagnostic and Statistical Manual of Mental Disorders (DSM-IV) – Fourth Edition],[29] attending the clinical services of the National Institute of Mental Health and Neuro Sciences (NIMHANS, Bangalore) and 16 “LR” healthy control subjects (group matched for age, sex, education and handedness) were recruited for the study. “High risk” in this study was defined to denote a subject without any psychiatric diagnosis but with a family history of schizophrenia in at least one of his/her sibling (i.e. brother or sister). “Low risk” was defined as absence of family history of psychiatric disorder in any of the first-degree relatives.

The DSM-IV diagnosis of schizophrenia in the affected sibling was established using the Mini International Neuropsychiatric Interview Plus (MINI Plus which has additional modules for diagnosing psychotic disorders[30]). The family history of psychiatric disorders in first-degree relatives (of both HR and LR subjects) was gathered using the Family Interview for Genetic Studies from at least two reliable adult informants in the family. None of the HR subjects had family history of psychiatric disorder (other than schizophrenia) in their first-degree relatives. None of the LR subjects had family history of any psychiatric disorder in their first-degree relatives.

### Clinical assessment of the study subjects

The study subjects (HR and LR) were assessed using the MINI Plus[30] to rule out any current or lifetime psychiatric disorder. All study subjects were right-handed as assessed by Annett Handedness Questionnaire.[31] None of the subjects (HR and LR) scored positive for alcohol use on CAGE questionnaire.[32] None had any other substance abuse or dependence. None of the subjects had any neurological disorder, mental retardation, history of significant head injury or any contraindication to MRI. After complete description of the study to the subjects, written informed consent was obtained from all subjects. The Institute’s ethics committee approved the study.

### Facial emotion recognition task

The subjects viewed the dynamic emotional expressions (short movie clips) that were derived from the TRENDS.[28] This is a recently developed and validated tool that is culturally appropriate for Indian subjects. From this tool, dynamic emotions of two men depicting fear, happy and neutral expressions were chosen. All faces were matched for overall luminosity and size, and were equally aligned on a black background. The stimuli were presented in a pseudo-random order, with each emotion expression (fear or happy) being immediately followed by a blank or neutral stimulus. The stimuli were presented using a projector (Sanyo ProX, Multiverse Project) and mirror system. The subjects received standardized and synchronized instructions to carefully attend to the stimulus and process the probable emotion that was expressed. They were instructed specifically not to verbalize/name (overt or covert) the emotions.

### Imaging procedures

#### Basic principles of fMRI

**Figure 1:**
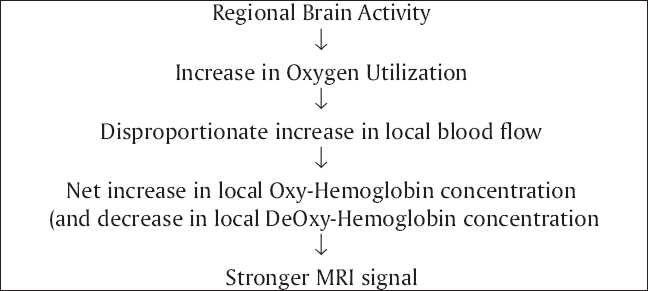
Flow chart diagram describing the basics of fMRI

The fMRI is a non-invasive imaging technique that is based upon the differential magnetization properties of the hemoglobin. Hemoglobin is diamagnetic when oxygenated but paramagnetic when deoxygenated. The magnetic resonance (MR) signal of blood will therefore differ depending on the level of oxygenation of hemoglobin. These differential signals can be detected using an appropriate MR pulse sequence as Blood Oxygen Level Dependent (BOLD) contrast. By collecting data in an MRI scanner with parameters sensitive to changes in magnetic susceptibility, one can assess changes in BOLD contrast. These changes can be either positive or negative depending upon the relative changes in both cerebral blood flow (CBF) and oxygen consumption. Increases in CBF that outstrip changes in oxygen consumption will lead to increased BOLD signal; conversely, decreases in CBF that outstrip changes in oxygen consumption will cause decreased BOLD signal intensity [Figure 1].[33,34]

#### Technical details of MRI and fMRI sequences

Magnetic Resonance Imaging (MRI) was done with 1.5-T Siemens Magnetom “vision” scanner (Siemens, Erlangen, Germany). Subjects were immobilized with cushions to avoid motion related artifacts. First, T1-weighted three-dimensional Magnetization Prepared Rapid Acquisition Gradient Echo sequence was performed to obtain detailed neuroanatomical information (TR=9.7 msec; TE=4 msec; nutation angle=12°; FOV=250 mm; slice thickness=1 mm; NEX=1; matrix=200×256; 160 sagittal slices). None of the scans showed any clinically significant structural abnormalities as verified by a neuroradiologist.

After obtaining the anatomical MR images, the images for the fMRI scan, namely, the echo-planar images (EPI) were obtained. They consisted of 144 functional acquisitions, with each acquisition consisting of 16 slices (slice thickness=8 mm without interslice gap) in axial plane covering the entire brain. The parameters for multi-shot EPI sequence using BOLD contrast were as follows: repetition time=4000 msec; echo time=76 msec; flip angle=90°; FOV=250 mm; matrix 128×128.

The dynamic emotional expression stimuli used for analyses in the present study included 12 happy, 12 fear, 12 neutral and blank projections (i.e. black background without any graphics or images to serve as absolute baseline) that were delivered in a pseudo-random order. Each stimulus (emotion expression or neutral stimulus or blank projection) was projected for 8 seconds.

### Statistical analyses

#### Analysis of socio-demographic/clinical data

The socio-demographic, clinical and region-of-interest (ROI) data were analyzed using the Statistical Package of Social Sciences, version 10 (SPSS Inc., Somers, NY, USA). The age as well as education years’ data were tested for normality using one-sample Kolmogorov–Smirnov test before conducting the parametric analyses. The data were found to be of normal distribution (*P*>0.05). The socio-demographic data were compared using the independent samples *t*-test and Chi-square test. The significance (two-tailed) was set at *P*<0.05.

#### fMRI analysis

The fMRI analysis was performed using Statistical Parametric Mapping-2 (SPM2) (http://www.fil.ion.ucl.ac.uk/spm). The EPI images were realigned and corrected for slice timing variations. The images were then normalized[35] to the Montreal Neurological Institute (MNI) space.[36] Finally, the images were smoothened with a Gaussian kernel of 6-mm full width at half maximum.

SPM2 combines the General Linear Model and Gaussian field theory to draw statistical inferences from BOLD response data regarding deviations from the null hypothesis in three-dimensional brain space.[37] The images were analyzed using a block design paradigm with canonical hemodynamic response function. Using random effects analyses, the differential hemodynamic responses (i.e. BOLD activity) between the HR and LR subjects during recognition of happy facial expression versus baseline as well as fearful facial expression versus baseline were examined through respective contrasts using a subtraction paradigm. The voxel-wise analysis produced a statistical parametric map of brain activation associated with facial emotion recognition task in the MNI space. Significance corrections for multiple comparisons were performed using a False Discovery Rate (FDR) correction (*P*<0.0125).[38]

Following this, ROI based analysis was performed to examine for amygdala abnormalities. Since the analysis was examining voxels contained in the selected gray matter regions (that were chosen based on the results of regional gray matter volume deficits obtained after group comparison analysis as described above), ROI based analysis was done along the lines of previous study.[26] This was performed using the small volume correction (SVC) function in SPM without correction for multiple comparisons in view of the *a priori* specification of this single brain region. The voxels included in the analysis were defined by a mask created for amygdale, using an automated software.[39] The coordinates of significant areas of activation were transformed from MNI space[36] into the stereotactic space of Talairach and Tournoux[40] using nonlinear transform.[41] The brain regions were localized from the Talairach and Tournoux coordinates using an automated software.[42]

## RESULTS

### Socio-demographic profile comparison between HR and LR subjects

HR and LR subjects did not differ significantly in age in years and number of years of education. The sex ratio was not significantly different between the two groups [Table 1]. All subjects were right-handed. Thus, the two groups (HR and LR subjects) were matched on age, sex, number of years of education and handedness.

**Table 1:** Comparison of demographic profile of high-risk and low-risk subjects

Variable	High-risk subjects (*n*=17)	Low-risk subjects (*n*=16)	Statistic	*P*
Age (years)*	25.2±4.2	24.4±3.7	t*=0.6	>0.5
Years of education*	13.2±2.1	13.3±3.3	t*=0.1	>0.5
Sex (M:F)**	14:3	14:2	*χ*^2^**=0.4	>0.5

* Comparison using independent samples *t*-test;

** Comparison using Chi-square test;

*P*>0.5, Not significantly different

### fMRI results

**Figure 2:**
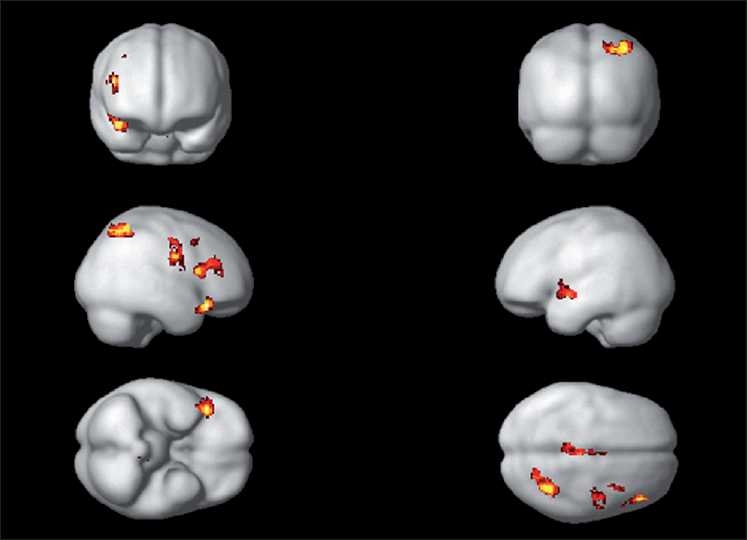
Rendered images depicting the brain regions of significantly deficient activation in HR subjects (*n*=17) in comparison to LR subjects (*n*=16). The deficient regions are highlighted in yellow- and red- representative of regions in Table 2

**Figure 3:**
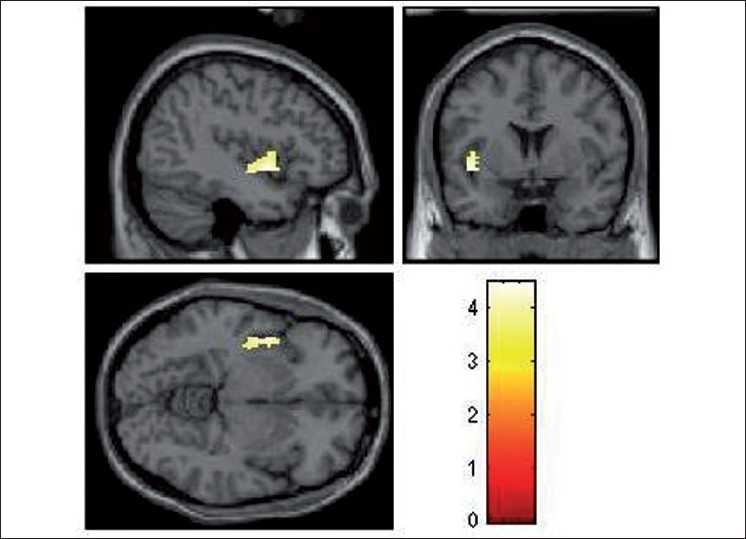
Rendered images depicting the significantly deficient activation in left insula in HR subjects (*n*=17) in comparison to LR subjects (*n*=16). The color bar is representative of the “T” scores given in Table 2

**Figure 4:**
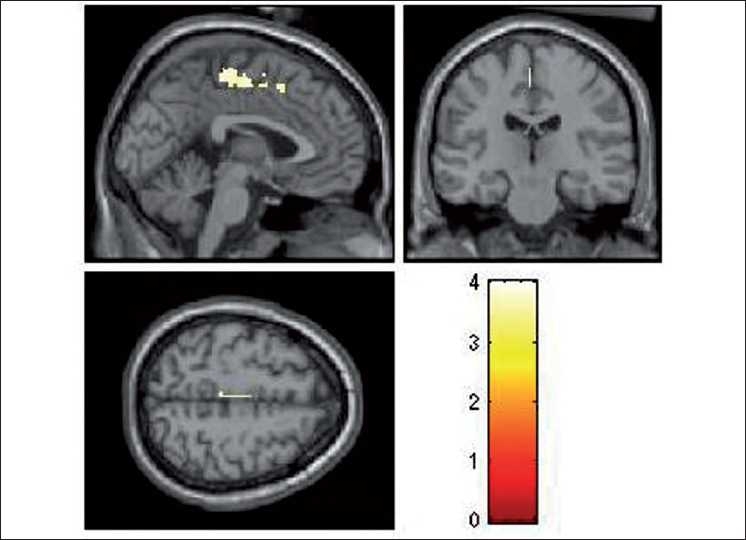
Rendered images depicting the significantly deficient activation in left medial frontal gyrus in HR subjects (*n*=17) in comparison to LR subjects (*n*=16). The color bar is representative of the “T” scores given in Table 2

**Figure 5:**
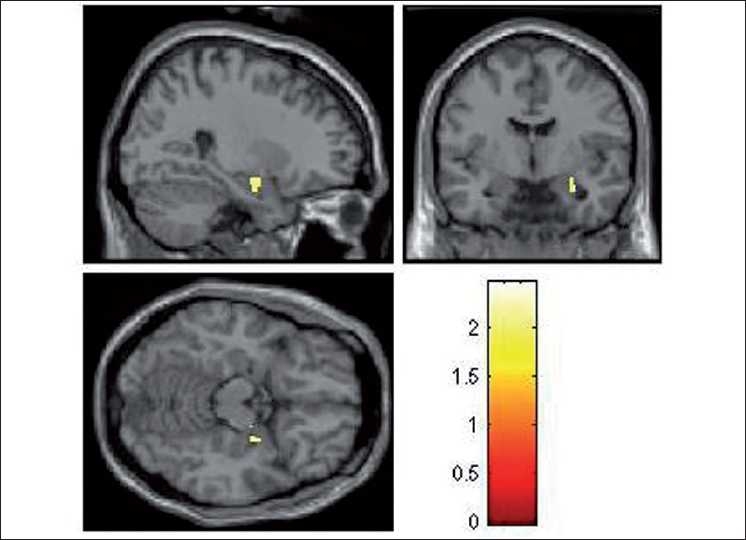
Rendered images depicting the significantly deficient activation in right amygdala in HR subjects (*n*=17) in comparison to LR subjects (*n*=16). The color bar is representative of the “T” score

During the emotional processing of fearful facial expression, HR subjects had significantly deficient activation of the following brain regions: left insula (limbic region), left medial frontal gyrus, right inferior frontal gyrus (orbito-frontal), right cingulate gyrus, right precentral gyrus and right inferior parietal lobule [Table 2; Figures 2–4]. In addition, ROI-based analysis revealed significantly deficient activation of right amygdala in HR subjects during fear processing with the following statistics (Talairach and Tournoux peak correlation coordinates: *x*=30, *y*=–3, *z*=–17; *T*=2.0; *P*=0.029) [Figure 5]. HR subjects did not differ significantly from LR subjects in brain activation during recognition of happy facial expressions.

**Table 2:** Brain regions with significantly deficient activation in high-risk subjects (*n*=17) in comparison to low-risk subjects (*n*=16)

Brain region	BA	Coordinates*			T	P**
		x	y	z		
Left insula (limbic cortex)	13	–42	4	6	4.5	<0.001
Left medial frontal gyrus	6	–1	–20	60	4.5	<0.001
Right inferior frontal gyrus (orbito-frontal)	47	52	28	–12	4.2	<0.001
Right anterior cingulate gyrus (rostral)	24	4	–2	44	4.6	<0.001
Right inferior parietal lobule	40	40	–50	56	4.8	<0.001
Right precentral gyrus	6	54	–6	34	5.6	<0.001

BA - Brodmann area;

* Alairach and Tournoux coordinates of peak difference;

** All scores significant (*P*<0.0125) after false discovery rate correction for multiple comparisons over the whole brain

## DISCUSSION

To the best of our knowledge, this is the first study to use dynamic facial emotion expressions to examine HR subjects for schizophrenia in comparison to age-, sex-, education- and handedness-matched LR subjects, using fMRI. Importantly, we used the emotional expression images that are culturally appropriate for Indian subjects, which have been validated in this population.[28] In this study, while processing the fearful emotional expression, HR subjects had significantly deficient activation of the following brain regions: left insula (limbic region), left medial frontal gyrus, left inferior frontal gyrus (i.e. orbito-frontal region), right cingulate gyrus, right precentral gyrus, right inferior parietal lobule and right amygdala [Table 2; Figures 2–5]. HR subjects did not differ significantly from LR subjects in brain activation during recognition of happy facial expressions.

### Abnormal emotional brain circuits and vulnerability to psychosis

Some of the findings of our study are in tune with the only previous fMRI study of similar nature (but this study had used methodologically sub-optimal static photographic images). Especially, the significant hypoactivation of amygdala that we have observed is in support of the previous study. Also, lack of differences in brain activation for positive emotional expression (i.e. happiness) with deficient activation specifically for negative emotional expression (i.e. fear) also is in tune with the previous study. Crucially, observation of these abnormalities using an “ecologically valid” and simple fMRI paradigm adds validity to our study findings. Moreover, such a differential abnormal recognition of negative emotions when compared to positive emotion has been reported in schizophrenia patients also.[43] Given this, it is plausible that our findings implicate that these brain activation deficits could indicate increased vulnerability to schizophrenia.

Importantly, our study observations suggest additional abnormalities in left insula, left inferior frontal gyrus (orbito-frontal region), right cingulate gyrus, right inferior parietal lobule as well as right precentral gyrus in HR subjects. The bilateral distribution of these deficits suggests that the emotional brain regional aberrations might be widespread in individuals at high risk for schizophrenia. Interestingly, in a recent comprehensive review report, all these brain regions have been reported to be crucial for processing facial emotion expression.[23]

The orbito-frontal cortex (OFC) is essential for integrating the emotional valence and self-monitoring so that the interpersonal as well as social behavior is adaptive.[44] Lesions of the OFC in humans have been shown to result in various social cognition abnormalities like apathy, social withdrawal, impaired identification of facial and vocal emotional expression, depressed mood, and flattened affect.[45,46] Most of these abnormalities share a striking similarity with the manifestations of negative syndrome in schizophrenia.

Similarly, the rostral component of the anterior cingulate gyrus is considered to be essential for emotion processing.[47] Moreover, this region has dense interconnections with other limbic brain regions, namely, the amygdala and the insular cortex. Hence, the widespread deficient activations in HR subjects involving theoretically relevant brain regions that are crucial for emotion processing strongly support “emotion-deficits” as an endophenotype for schizophrenia.

### Schizophrenia: Emotion processing, theory of mind and mirror neurons

A novel aberration identified in our study is the significantly deficient activation in right inferior parietal lobule as well as in right precentral gyrus. Interestingly, these brain regions have been reported to be deficient in various structural as well as functional neuroimaging studies in schizophrenia.[48–50] Especially, the inferior parietal lobule is considered to be an important region for the “theory of mind” functions.[51] Theory of mind is among the key constituents of “social cognition”[52] and the inferior parietal lobule is a critical component of the neural circuits that sub-serve all these constituents of social cognition.[51] The “social brain hypothesis of schizophrenia”, which conceptualizes schizophrenia as primarily a disorder of social cognition, has been proposed as a useful heuristic to understand the intriguing clinical manifestations of schizophrenia.[53]

In an influential review on the role of parietal cortex in schizophrenia,[51] it has been suggested that the parietal cortex abnormalities might result in self-monitoring deficits in these patients. Interestingly, it has been argued that attentional dysfunctions and certain impairments of motor control and motor imagery all point toward the involvement of the parietal cortex in the disorder. In this context, concurrent parietal as well as motor (i.e. precentral gyrus) cortical deficient activations elucidated in this study become significant. Such a relationship becomes especially more relevant given the recent fMRI study observations that theory of mind and face-to-face emotional interactions are interlinked with an involvement of these brain regions.[54] Also, these functions were shown to be associated with mirror neurons.[54]

“Mirror neurons” provide an understanding of social cognition at the cellular level.[55] Mirror neurons activate when the subject observes goal-directed action in another individual. Thus, they serve to mirror or simulate observed intentional actions within the motor cortex of the observer – they internally “represent” an action.[55] Importantly, in addition to various brain regions, mirror neurons are present in motor cortex also. Recent neuroimaging studies examining the functions of mirror neurons postulate that their aberrant activation may form the basis of various psychotic experiences. Indeed, an extensive review on “mirror neuron hypothesis on schizophrenia” argues for such a possibility.[56] Given this, our findings might suggest a useful heuristic that mirror neuron abnormalities might be linked to the emotion processing deficits in HR subjects as well as probably in schizophrenia patients also. Since, mirror neurons are considered to be of evolutionarily recent origin, this proposed relationship might offer support to the “evolutionary theories of schizophrenia”, that are postulated as an overarching and unifying hypotheses to understand this complex neuropsychiatric disorder.[53] However, this requires to be explored by systematic research using fMRI studies employing relevant paradigms to assess the inter-relationship between emotion processing, theory of mind and mirror neurons in HR subjects as well as in schizophrenia patients.

### Methodological issues

This is the first study to use dynamic facial emotion expressions to examine HR subjects for schizophrenia in comparison to age-, sex-, education- and handedness-matched LR subjects, using functional MRI. Some of the significant methodological advantages of the study include the following: 1) use of dynamic facial expressions; 2) utilizing a simple and ecologically valid “implicit” fMRI paradigm with less demand on performance by the subjects during the scan; 3) use of validated and culturally appropriate images (i.e. TRENDS); 4) structured interview to assess the clinical status of the study subjects; 5) age-, sex-, education-, handedness-matched healthy controls; 6) use of methodologically superior and validated software for fMRI analysis (SPM2); 7) rigorous statistical tests for fMRI using random effects analysis; 8) robust correction using a conservative threshold (false discovery rate threshold of *P*<0.0125) to correct for multiple comparison statistical analyses (and thus avoiding spurious findings); and 9) sample size of HR subjects is comparatively larger than the previous study; also, this sample size is optimally powerful as proposed by the influential reports in fMRI methodology.[57]

However, the following factors might be construed as potential limitations of our study. The accuracy of emotion recognition in the subjects was not systematically assessed by recording some “explicit” intra-scanner response of the subjects. While this might be considered as a limitation, we wanted to avoid additional cognitive/motor components of brain function that might interfere or potentially confound the brain activation patterns. Thus, we deliberately avoided this to make the paradigm simpler and ecologically valid. Now that the “implicit” emotion-processing paradigm has demonstrated important findings, future studies should aim at examining the interplay of these additional factors in emotion processing. We assessed only two emotions – happy and fear – mainly to restrict the duration of fMRI scan time within optimal limits. These two emotions were specifically chosen because of their differential nature of aberrance in schizophrenia as well as their simplicity. With the availability of the advanced 3-T scanner, significantly faster imaging is possible; ongoing studies at NIMHANS employ faster event-related fMRI to assess additional emotions also within short scan time.

## CONCLUSIONS AND FUTURE DIRECTIONS

In conclusion, this is the first study to use dynamic facial emotion expressions to examine HR subjects for schizophrenia in comparison to age-, sex-, education- and handedness-matched LR subjects, using functional MRI. Study findings suggest neurohemodynamic abnormalities involving frontal and limbic cortices that are potential indicators for increased vulnerability toward schizophrenia. These findings support the concept of “emotion processing deficit as an endophenotype in schizophrenia”. Also, these findings can be theoretically linked to theory of mind as well as probable mirror neuron abnormalities in schizophrenia. Some of our novel findings require replication as well as there is a compelling need to extend this line of research using more advanced paradigms. Also, the clinical utility of these novel findings in predicting the development of psychosis needs to be evaluated in longitudinal studies of subjects at high risk for schizophrenia.

## References

[1] Schultz SK, Andreasen NC (1999). Schizophrenia. Lancet.

[2] Cutting J (1981). Judgment of emotional recognition expression in schizophrenics. Br J Psychiatry.

[3] Schneider F, Gur RC, Gur RE, Shtasel DL (1995). Emotional processing in schizophrenia: Neuro behavioural probes in relation to psychopathology. Schizophr Res.

[4] Bediou B, Krolak-Salmon P, Saoud M, Henaff MA, Burt M, Dalery J (2005). Facial expression and sex recognition in schizophrenia and depression. Can J Psychiatry.

[5] Mandal MK, Pandey R, Prasad AB (1998). Facial expressions of emotions and schizophrenia: A review. Schizophr Bull.

[6] Kerr S, Neale J (1993). Emotion perception in schizophrenia: Specific deficit or further evidence of generalised poor performance?. J Abnorm Psychol.

[7] Salem J, Kring A, Kerr S (1996). More evidence for generalised poor performance in facial emotion perception in schizophrenics. J Abnorm Psychol.

[8] Gessler S, Cutting J, Frith C, Weinman J (1989). Schizophrenic inability to judge facial emotion: A controlled study. Br J Clin Psychol.

[9] Kucharska-Pietura K, David AS (2005). Perception of facial and vocal affect by people with schizophrenia in early and late stages of illness. Br J Psychiatry.

[10] Herbener ES, Hill SK, Marvin RW, Sweeney JA (2005). Effects of antipsychotic treatment on emotion perception deficits in first-episode schizophrenia. Am J Psychiatry.

[11] Borod J, Alpert M, Brozgold A, Martin C (1993). A preliminary comparison of flat affect schizophrenics and brain-damaged patients on measures of affective processing. J Commun Disord.

[12] Penn DL, Combs DR, Ritchie M, Francis J, Cassisi J, Morris S (2000). Emotion recognition in schizophrenia: Further investigation of generalized versus specific deficit models. J Abnorm Psychol.

[13] Silver H, Shlomo N, Turner T, Gur RC (2002). Perception of happy and sad facial expressions in chronic schizophrenia: Evidence for two evaluative systems. Schizophr Res.

[14] Hall J, Harris J, Sprengelmeyer R, Sprengelmeyer A, Young A, Santos I (2004). Social cognition and face processing in schizophrenia. Br J Psychiatry.

[15] Heimberg C, Gur R, Erwin R, Shtasel D, Gur R (1992). Facial emotion discrimination: III. Behavioral findings in schizophrenia. Psychiatry Res.

[16] Kee KS, Green MF, Mintz J, Brekke JS (2003). Is emotion processing a predictor of functional outcome in schizophrenia?. Schizophr Bull.

[17] Mueser KT, Penn DL, Blanchard JJ, Bellack AS (1997). Affect recognition in schizophrenia: A synthesis of findings across three studies. Psychiatry.

[18] Edwards J, Jackson HJ, Pattison PE (2002). Emotion recognition via facial expression and affective prosody in schizophrenia: A methodological review. Clin Psychol Rev.

[19] McCownW, Johnson J, Austin S, Shefsky M (1988). Deficits in ability to decode facial affects in families of schizophrenics. Psychother. Priv. Pract.

[20] Toomey R, Sideman LJ, Lyons MJ, Faraone SV, Tsuang MT (1999). Poor perception of nonverbal social-emotional cues in relatives of schizophrenic patients. Schizophr Res.

[21] Bediou B, Asri F, Brunelin J, Krolak-Salmon P, D’Amato T, Saoud M (2007). Emotion recognition and genetic vulnerability to schizophrenia. Br J Psychiatry.

[22] Ekman P, Friesen W (1976). Pictures of Facial Affect.

[23] Vuilleumier P, Pourtois G (2007). Distributed and interactive brain mechanisms during emotion face perception: Evidence from functional neuroimaging. Neuropsychologia.

[24] Aleman A, Kahn RS (2005). Strange feelings: Do amygdala abnormalities dysregulate the emotional brain in schizophrenia?. Prog Neurobiol.

[25] van Rijn S, Aleman A, Swaab H, Kahn RS (2005). Neurobiology of emotion and high risk for schizophrenia: Role of the amygdala and the X-chromosome. Neurosci Biobehav Rev.

[26] Habel U, Klein M, Shah NJ, Toni I, Zilles K, Falkai P (2004). Genetic load on amygdala hypofunction during sadness in nonaffected brothers of schizophrenia patients. Am J Psychiatry.

[27] Hunter MD, Farrow TF, Papadakis NG, Wilkinson ID, Woodruff PW, Spence SA (2003). Approaching an ecologically valid functional anatomy of spontaneous “willed” action. Neuroimage.

[28] Rishikesh B, Raghunandan VN, Venkatasubramanian G, Subbakrishna DK, Jayakumar PN, Gangadhar BN TRENDS – Tool for Recognition of Emotions in Neuropsychiatric Disorders.

[29] (1994). American Psychiatric Association. DSM-IV: Diagnostic and Statistical Manual of Mental Disorders.

[30] Sheehan DV, Lecrubier Y, Harnett-Sheehan K, Amorim P, Janavs J, Weiller E (1998). The Mini International Neuropsychiatric Interview (M.I.N.I.): The development and validation of a structured diagnostic psychiatric interview. J Clin Psychiatry.

[31] Annett M (1967). The binomial distribution of right, mixed and left handedness. Q J Exp Psychol.

[32] Ewing JA (1984). Detecting alcoholism. The CAGE Questionnaire. JAMA.

[33] Heeger DJ, Ress D (2002). What does fMRI tell us about neuronal activity?. Nat Rev Neurosci.

[34] http://www.en.wikipedia.org/wiki/Functional_magnetic_resonance_imaging.

[35] Friston KJ, Ashburner J, Frith CD, Poline JB, Heather JD, Frackowiak RSJ (1995). Spatial registration and normalization images. Hum Brain Mapp.

[36] Evans AC, Collins DL, Mills SR, Brown ED, Kelly RL, Peters TM (1993). 3D statistical neuroanatomical models from 305 MRI volumes. IEEE Nucl Sci Symp Medical Imaging Conf Proc.

[37] Holmes A, Poline JB, Friston KJ, Frackowiak RS, Friston KJ, Frith CD, Dolan RJ, Mazziotta JC (1997). Characterizing brain images with the general linear model. Human Brain Function.

[38] Genovese CR, Lazar NA, Nichols T (2002). Thresholding of statistical maps in functional neuroimaging using the false discovery rate. Neuroimage.

[39] Maldjian JA, Laurienti PJ, Kraft RA, Burdette JH (2003). An automated method for neuroanatomic and cytoarchitectonic atlas-based interrogation of fMRI data sets. Neuroimage.

[40] Talairach P, Tournoux JA (1988). A Stereotactic Co-Planar Atlas of Human Brain.

[41] Brett M, Johnsrude IS, Owen AM (2002). The problem of functional localization in the human brain. Nat Rev Neurosci.

[42] Lancaster JL, Woldorff MG, Parsons LM, Liotti M, Freitas CS, Rainey L (2000). Automated Talairach atlas labels for functional brain mapping. Hum Brain Mapp.

[43] An SK, Lee E, Kim JJ, Namkoong K, Kang JI, Jeon JH (2006). Greater impairment in negative emotion evaluation ability in patients with paranoid schizophrenia. Yonsei Med J.

[44] Beer JS, John OP, Scabini D, Knight RT (2006). Orbitofrontal cortex and social behavior: Integrating self-monitoring and emotion-cognition interactions. J Cogn Neurosci.

[45] Rolls ET (1996). The orbitofrontal cortex. Philos Trans R Soc Lond B Biol Sci.

[46] Hornak J, Bramham J, Rolls ET, Morris RG, O’Doherty J, Bullock PR (2003). Changes in emotion after circumscribed surgical lesions of the orbitofrontal and cingulate cortices. Brain.

[47] Mohanty A, Engels AS, Herrington JD, Heller W, Ho MH, Banich MT (2007). Differential engagement of anterior cingulate cortex subdivisions for cognitive and emotional function. Psychophysiology.

[48] Ganesan V, Hunter MD, Spence SA (2005). Schneiderian first-rank symptoms and right parietal hyperactivation: A replication using FMRI. Am J Psychiatry.

[49] Jayakumar PN, Venkatasubramanian G, Gangadhar BN, Janakiramaiah N, Keshavan MS (2005). Optimized voxel-based morphometry of gray matter volume in first-episode, antipsychotic-naïve schizophrenia. Prog Neuropsychopharmacol Biol Psychiatry.

[50] Spence SA (2002). Alien motor phenomena: A window on to agency. Cogn Neuropsychiatry.

[51] Danckert J, Saoud M, Maruff P (2004). Attention, motor control and motor imagery in schizophrenia: Implications for the role of the parietal cortex. Schizophr Res.

[52] Grady CL, Keightley ML (2002). Studies of altered social cognition in neuropsychiatric disorders using functional neuroimaging. Can J Psychiatry.

[53] Burns J (2006). The social brain hypothesis of schizophrenia. World Psychiatry.

[54] Schulte-Ruther M, Markowitsch HJ, Fink GR, Piefke M (2007). Mirror neuron and theory of mind mechanisms involved in face-to-face interactions: A functional magnetic resonance imaging approach to empathy. J Cogn Neurosci.

[55] Gallese V, Keysers C, Rizzolatti G (2004). A unifying view of the basis of social cognition. Trends Cogn Sci.

[56] Arbib MA, Mundhenk TN (2005). Schizophrenia and the mirror system: An essay. Neuropsychologia.

[57] Friston KJ, Holmes AP, Worsley KJ (1999). How many subjects constitute a study?. Neuroimage.

